# RNF8-Independent Lys63 Poly-Ubiquitylation Prevents Genomic Instability in Response to Replication-Associated DNA Damage

**DOI:** 10.1371/journal.pone.0089997

**Published:** 2014-02-28

**Authors:** Chantal H. M. A. Ramaekers, Twan van den Beucken, Robert G. Bristow, Roland K. Chiu, Daniel Durocher, Bradly G. Wouters

**Affiliations:** 1 Ontario Cancer Institute and Campbell Family Institute for Cancer Research, Princess Margaret Cancer Centre, University Health Network, Toronto, Ontario, Canada; 2 Maastricht Radiation Oncology (MaastRO) Lab, GROW – School for Oncology and Developmental Biology, Maastricht University, Maastricht, The Netherlands; 3 Departments of Radiation Oncology and Medical Biophysics, University of Toronto, Toronto, Ontario, Canada; 4 Department of Radiation Oncology, University Medical Center Groningen, University of Groningen, Groningen, The Netherlands; 5 Samuel Lunenfeld Research Institute, Mount Sinai Hospital, Toronto, Ontario, Canada; 6 Department of Molecular Genetics, University of Toronto, Toronto, Ontario, Canada; University of Minnesota, United States of America

## Abstract

The cellular response to DNA double strand breaks (DSBs) involves the ordered assembly of repair proteins at or near sites of damage. This process is mediated through post-translational protein modifications that include both phosphorylation and ubiquitylation. Recent data have demonstrated that recruitment of the repair proteins BRCA1, 53BP1, and RAD18 to ionizing irradiation (IR) induced DSBs is dependent on formation of non-canonical K63-linked polyubiquitin chains by the RNF8 and RNF168 ubiquitin ligases. Here we report a novel role for K63-ubiquitylation in response to replication-associated DSBs that contributes to both cell survival and maintenance of genome stability. Suppression of K63-ubiquitylation markedly increases large-scale mutations and chromosomal aberrations in response to endogenous or exogenous replication-associated DSBs. These effects are associated with an S-phase specific defect in DNA repair as revealed by an increase in residual 53BP1 foci. Use of both knockdown and knockout cell lines indicates that unlike the case for IR-induced DSBs, the requirement for K63-ubiquitylation for the repair of replication associated DSBs was found to be RNF8-independent. Our findings reveal the existence of a novel K63-ubiquitylation dependent repair pathway that contributes to the maintenance of genome integrity in response to replication-associated DSBs.

## Introduction

To maintain genomic stability mammalian cells have evolved extensive signalling and repair networks that respond to DNA damage. DNA double strand breaks (DSBs) are considered the most cytotoxic lesions and their inaccurate repair can lead to mutation, chromosomal translocation and tumorigenesis [1], [2]. DSBs are caused by both exogenous agents and endogenous processes, including ionizing radiation (IR) and collapsed replication forks, respectively. Cells respond to DSBs through sequential recruitment of various signalling and repair proteins, many of which can be visualized as discrete foci at sites of damage [3], [4].

Although many protein-protein interactions in repair signalling depend on phosphorylation and phospho-binding domains, an important role has also emerged for protein ubiquitylation. In the case of IR-induced direct two-ended DSBs, the ubiquitin (Ub) ligases RNF8 and RNF168 are required for the recruitment of essential downstream repair proteins including BRCA1, 53BP1, and RAD18 [5], [6], [7], [8], [9], [10], [11], [12]. RNF8 binds to phosphorylated MDC1 at DSBs through its FHA domain and together with UBC13, ubiquitylates histones H2A and H2AX. RNF8-UBC13 catalyses the formation of a polyUb chain, in which successive Ub molecules are linked through lysine 63 (K63-ubiquitylation). Unlike K48-ubiquitylation, which promotes protein degradation, K63-ubiquitylation promotes protein interactions [13], [14]. K63-ubiquitylation of H2A and H2AX facilitate recruitment of RNF168 through its MIU (motif interacting with ubiquitin) domains [10], [11]. RNF168 is encoded by the gene mutated in RIDDLE syndrome and functions to further amplify K63-ubiquitylation of histones and possibly other substrates [10], [11]. RNF8 and RNF168 dependent K63-ubiquitylation mediates recruitment of the RAP80-ABRA1-BRCA1 complex and the accumulation of 53BP1 to DNA lesions [5], [6], [7], [8], [10], [11]. RNF8 dependent K63-ubiquitylation also promotes binding of RAD18, which in turn mediates recruitment of the homologous recombination (HR) factor RAD51C [12]. Defects in RNF8 or RNF168 impair K63-ubiquitylation and recruitment of these essential DNA repair factors resulting in increased sensitivity to IR-induced DSBs.

In this study we investigated the importance of K63-ubiquitylation in DSB-repair during S-phase. Recently, it has become clear that, besides IR-induced two-ended DSBs produced in G2-phase, secondary replication-associated DSBs in S-phase are a major substrate for HR-dependent repair following IR [15]. These secondary DSBs are the result of damage encountered by ongoing replication forks thereby leading to their collapse. This type of DSB also occurs when endogenously produced single strand breaks (SSBs) arrive at replication forks, resulting in fork run-off [16]. Their repair is also critical for maintenance of genome stability and for the response to several S-phase specific chemotherapeutic agents. This includes the topoisomerase I poison camptothecin (CPT), which exerts its cytotoxic effect by stabilizing a SSB intermediate and greatly increasing the number of collapsed forks during replication [17], [18], [19], as well as inhibitors of poly ADP-ribose polymerase (PARP), which similarly increase SSBs that collide with replication forks [20], [21], [22]. Here, we report that K63-ubiquitylation plays a crucial and unique role in the repair of such replication-associated DNA DSBs. Partial disruption of K63-ubiquitylation was achieved through stable transgene expression of a Ub allele harbouring a K63R mutation. The resulting ‘hypomorphic’ allele revealed a unique phenotype demonstrating the remarkable dependence on K63-ubiquitylation for accurate repair of DNA double strand breaks that occur in S-phase of the cell cycle. Disruption of K63-ubiquitylation resulted in a dramatic increase in genetic instability and an increased sensitivity to replication-associated DSBs. The DNA repair defect observed after suppression of K63-ubiquitylation is characterized by an increase in S-phase specific damage and residual 53BP1 foci. Furthermore, we provide strong genetic evidence using RNF8 knockout cells that in contrast to IR-induced DSBs, K63-ubiquitylation required to confer resistance to damage during S-phase is largely RNF8 independent.

## Materials and Methods

### Cell culture and transfection

Construction of WTUb-GFP and K63RUb-GFP expression plasmids has been described previously [23]. For this study we regenerated previously described cell-lines [24]. A549 (human epithelial lung carcinoma cells) from ATCC were used or cells were cotransfected with WTUb or K63RUb plasmids and a pBabePuro plasmid using FuGene 6 (Roche, Basel, Zwitserland) and stable expressing cells were selected in 1 µg/ml puromycin (Sigma). High expressing cells were sorted for by flow cytometry based on GFP expression (FACSAria, BD Biosciences Pharmingen, San Diego, California, USA). Cells were cultured in DMEM (Invitrogen) supplemented with 10% FBS (PAA), 1 mM SodiumPyruvate (Gibco) at 37°C and 5% CO_2_. Primary WT MEFs and same litter RNF8-/- MEFs were generously provided by R. Hakem [25]. MEFs were immortalized by 3T3 passaging and cultured similar as described above, medium was additionally supplemented with 100 nM β-mercaptoethanol.

### Plasmids and lentiviral work

WT-Ub-GFP and K63R-Ub-GFP inserts were PCR amplified from the original vectors and cloned as Age1/EcoR1 fragments into lentiviral vector pJLM1. The following lentiviral shRNA constructs were used for knockdown: TRCN0000007216 (UBC13), TRCN0000015555 (SHPRH), TRCN0000272562 (HLTF), TRCN0000003438 (RNF8). Lentiviral particles were generated by co-transfection of 293T cells with packaging plasmids pCMVdR8.74psPAX2 and pMD2.G together with shRNA vector pLKO.1 or pLJM1. Virus supernatant was harvested 48 and 72 hrs post transfection. A549 cells or MEFs were transduced overnight with lentiviral supernatant in the presence of 8 µg/ml polybrene. Infected cells were grown in 2 µg/ml puromycin containing media for 2 days to select for stable expressing pools.

### siRNA

The following short interfering RNA (siRNA) duplexes were purchased from Sigma targeting the indicated gene products: RNF8-si; 5′-GGA CAA UUA UGG ACA ACA AdTdT-3′, RAP80-si; 5′- UUG UGA AGC AGG UAC AGA GUU UCC CdTdT-3′. Stealth RNAi Negative Control Med GC was ordered from Invitrogen (12935-300). For siRNA experiments, cells were double transfected at 90% confluency, 72 and 24 hrs before start of the experiment, with the indicated siRNA's at a final concentration of 100 nM using oligofectamine (Invitrogen).

### RNA extraction and quantitative RT-PCR

RNA was isolated using Tri reagent (Sigma) according to manufacturers' instructions. RNA samples were reverse transcribed using q-Script kit as described by manufacturer (Quantas). Real-time PCR was performed on an Eppendorf Realplex^2^ mastercycler using SYBR green (Quantas). The following Q-PCR primers were used: RNF8 F_5′-TGC TAG AGA ATG AGC TCC AAT G-3′; RNF8 R_5′-CGC ACT ACC TGG CAG TCT TT-3′; RAP80 F_5′-AGG TAT CCT GCC CGC TAT GT-3′; RAP80 R_5′-TCA CTC TTG GTC TTG GCC TC-3′; UBC13 F_5′-AGC CCA GAC ATC TTC AGT CC-3′; UBC13 R_5′- TAA ACC AGG ATG GGG GAA AT-3′.

### Clonogenic survival assays

Cells were seeded (range 200 to 5000 cells) in triplicate in 60-mm dishes either directly in DMEM or DMEM containing the indicated concentrations of PARP-inhibitor KU0058948. For CPT-assays cells were seeded to attach overnight before 24 hrs CPT treatment. For IR-assays, 80% confluent cells were irradiated with the indicated doses using a Cesium 137 source (GC-40E Nordion, dose-rate 0.83 Gy/min), 1 hr after IR cells were seeded similar to described above. Cells were incubated for 14 days to obtain colony formation. Resulting colonies were fixed and stained using 2% bromophenol blue in 80% ethanol, colonies containing ≥ 50 cells were counted. All experiments were normalized for plating efficiency. DNA-PK inhibitor KU0057788 treatment was always started 1 h before additional treatments as indicated.

### Proliferation assays

Cells were plated at a low density (10% confluence) in 6-well plates at least in duplicate and left to attach overnight. Cells were incubated at 37°C, 5% CO_2_ and growth was monitored using an automated microscope (IncuCyte, Essen Instruments, Inc., Michigan, USA).

### Mutation frequency assays

*HPRT* mutant- free cells were selected for by culturing cells in DMEM supplemented with hypoxanthine, aminopterin and thymidine (HAT) for 1 week. Cells were seeded (1x10^6^) in 100-mm dishes and cultured for 7 or 14 days in regular DMEM (spontaneous mutations) or continuous treatments were started the following day by adding DMEM containing PARP-inhibitor KU0058948 (0, 1 µM) or CPT (0, 5 and 20 nM) or cells were irradiated (0, 1, and 4 Gy) and cultured for 6 additional days. Subsequently, cells (4x10^5^) were seeded on 100-mm dishes in DMEM containing 30 µM 6-thioguanine (6-TG) and incubated for 14 days to obtain colony formation. In parallel, cells were plated (200) in regular DMEM to assess plating efficiency. Colonies were fixed and stained using 2% bromophenol blue in 80% ethanol, colonies containing ≥ 50 cells were counted. DNA-PK inhibitor KU0057788 treatment was started 1 h before additional treatments as indicated.

### Mutation spectra analyses

IR-induced mutated *HPRT* clones were obtained by seeding 1x10^5^ HAT-selected cells in 35-mm dishes, individual dishes were irradiated (6 Gy) 24 hrs later. After subculturing the cells for 6 days, from each 35-mm dish 1x10^5^ cells were re-seeded in 35-mm dishes in medium containing 30 µM 6-TG. CPT-induced mutants were obtained in a similar way as described for IR-induced mutants except cells were treated with 20 nM CPT for 6 days. For spontaneous mutants 4x10^5^ cells were seeded per 100-mm dish in 6-TG medium. One colony per dish was picked to avoid sister clones and grown until enough cells were obtained to isolate total RNA (TRI-reagent, Sigma) and genomic DNA (genomic DNA isolation kit, Norgen) according to the manufacturer's protocols. The HPRT gene was subjected to RT-PCR, followed by sequencing using the following overlapping primers: HPRT1 F_5′-CTT CCT CCT CCT GAG CAG TC-3′; HPRT2 R_5′-AAG CAG ATG GCC ACA GAA CT-3′; HPRT3 F_5′-CCT GGC GTC GTG ATT AGT G-3′; HPRT4 R_5′-TTT ACT GGC GAT GTC AAT AGG A-3′; HPRT5 F_5′-GAC CAG TCA ACA GGG GAC AT-3′; and HPRT6 R_5′-ATG TCC CCT GTT GAC TGG TC-3′ [24]. In case there was no cDNA product obtained, individual HPRT exons1-9 were PCR amplified at the genomic DNA level, using the following primers:

Exon 1 F_5′- GCT CCG CCA CCG GCT TCC TCC-3′, Exon 1 R_5′-GCC GAA CCC GGG AAA CTG G-3′, Exon 2 F_5′-TGT AAT GCT CTC ATT GAA ACA GC-3′, Exon 2 R_5′-AAG GCC CTC CTC TTT TAT TTT T-3′, Exon 3 F_5′-TTC CCA CCT CAC CTC TCA AG-3′, Exon 3 R_5′-TGG TTT GCA GAG ATT CAA AGA A-5′, Exon 4 F_5′-TCA GTA ATG GCC GAT TAG GAC-3′, Exon 4 R_5′-AGT CCC ACA GAG GCA GAC AG-3′, Exon 5 F_5′-GAA ATA CCG TTT TAT TCA TTG TAC TG-3′, Exon 5 R_5′-TGT GAA CTT ACT TCC ACA ATC AAG A-3′, Exon 6 F_5′-GAA GGA CAA CAT CAT AAC TCC CTA A-3′, Exon 6 R_5′-CTG CCA TGC TAT TCA GGA CA-3′, Exon 7 F_5′-AAC AGC TTG CTG GTG AAA AG-3′, Exon 7 R_5′-TCT GGC TTA TAT CCA ACA CTT CG-5′, Exon 8 F_5′-TTT TTG TCA ATC ATT TAA CCA TCT TT-5′, Exon 8 R_5′-CAT ATC AAA GTG GGA GGC CAG T-3′, Exon 9 F_5′-GCT ACA GTG AGC CAA CAT CAC G-3′, Exon 9 R_5′-CTG CTG ACA AAG ATT CAC TGG-3′.

### Antibodies and western blot analysis

We employed the following antibodies: rabbit anti-UbK63 chains (Millipore), mouse anti-ubiquitin (Chemicon), mouse anti-γH2AX (clone JBW301, Upstate), rabbit anti-γH2AX (Epitomics), rabbit anti-53BP1 (Alexis). Following the indicated treatments whole cell lysates were prepared using lysis buffer containing: 50 mM Tris-HCl [pH 7.4], 150 mM NaCl, 1% NP-40, 0.5% Sodium Deoxycholate, 1 mM EDTA, 0.1% SDS, supplemented with protease- (EDTA-free Complete tablets, Roche) and phosphatase inhibitors (PhosSTOP tablets, Roche). Supernatants were boiled in Laemmli buffer and proteins were resolved by SDS-PAGE. Proteins were transferred onto PVDF membranes and blocked for 1 hour in 5% skim milk in TBS, 0.05% Tween-20 (TBS-T). Membranes were probed overnight at 4°C with antibodies directed against K63 chains (1∶1000) or ubiquitin (1∶500). Bound antibodies were visualized using HRP-linked secondary antibodies (anti-rabbit (GE Healthcare) and anti-mouse (GE Healthcare)) and ECL luminescence (Pierce).

### Immunofluorescence microscopy

To visualize foci cells were grown on coverslips and treated as indicated. Cells were fixed with 2% para-formaldehyde, 0.2% Triton X-100 in PBS, pH 8.2 for 20 min. at room temperature. Subsequently washed with PBS and treated with 0.5% NP-40 for 20 min. at room temperature. Cells were washed with PBS before blocking with 2% BSA, 1% donkey serum in PBS for 1 hr at room temperature. Samples were incubated with primary antibodies anti-γH2AX (1∶800), anti-53BP1 (1∶1000). Antibodies were diluted in 3% BSA in PBS and incubated overnight at 4°C. Cells were washed with 0.5% BSA, 0.175% Tween20 in PBS and then stained with Alexa Fluor 568 donkey anti-mouse/rabbit IgG, Alexa Fluor 647 donkey anti-mouse/rabbit IgG (Molecular Probes) for 45 min. at room temperature. Secondary antibodies were diluted 1∶500 in 3% BSA in PBS. DNA was counterstained with DAPI (0.1 µg/ml) and mounted with Vectashield (Vector Laboratories). S-phase cells were labelled using EdU-labeling kit Alexa Fluor 647 (Click-iT EdU assays, Molecular Probes, Invitrogen) according to manufacturer's protocol. Confocal or widefield three-dimensional images were visualized using Olympus IX81 inverted microscope fitted with a Disk Scanning Unit (DSU), equipped with PLAPON 60X 1.42NA or UPLSAPO 100X 1.4NA oil-immersion objectives and a 16-bit Photometrics Cascade 512B EM-CCD camera (Roper Scientific, Tuscon, AZ). Z-stacks, 55 planes, 0.29 micron were acquired using In Vivo Software (Media Cybernetics, Bethesda, MA) or MetaMorph software and in some cases computationally deconvolved using 25 iterations of 3D Blind deconvolution (Autoquant, Media Cybernetics). Total intensity or deconvolved images were analysed using Image Pro Analyzer (Media Cybernetics).

### Statistical Analysis

Unpaired Student's *t* test was used to test significance between populations, *p*<0.05 was considered significant (indicated by * in the figures). Points and error bars plotted in the graphs of all figures represent the mean ± standard deviation (sd.) or standard error of the mean (s.e.m.) as indicated.

## Results

### K63-ubiquitylation is required to maintain genome integrity

**Figure 1:**
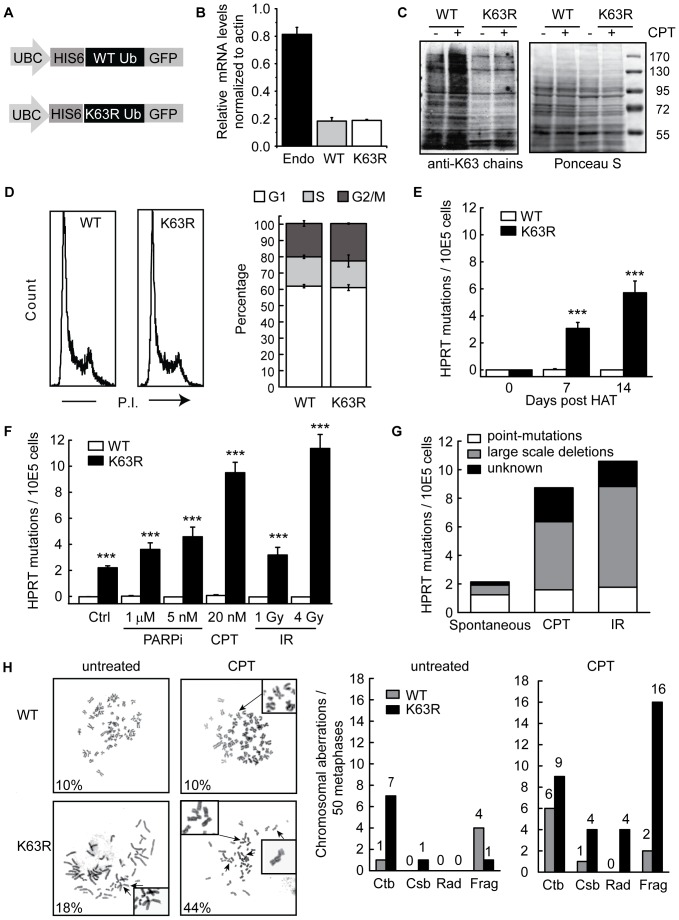
K63RUb expression induces genomic instability. (A) Schematic overview of WTUb and K63RUb expression constructs. Expression of a 6xHis-tagged WT or K63R ubiquitin B-GFP fusion protein is driven by the UBC promoter. (B) mRNA expression levels of endogenous ubiquitin B and expressed 6xHis-tagged WT or K63R ubiquitin B determined by real-time PCR. (C) Impaired K63-linked ubiquitin chain formation, in untreated or CPT (1h, 100nM) treated A549 K63RUb cells, confirmed by comparison to K63 ubiquitin chain abundance in A549 WTUb cells by Western blot using a K63-chain specific antibody. Ponceau S staining indicates equal loading. (D) Cell cycle distribution of normal cycling A549 WTUb and K63RUb overexpressing cells determined by FACS analysis. Data are mean of 3 independent exp's ± sd. (E-F) Mutations at the *HPRT* locus analysed in WTUb and K63RUb *HPRT* mutant-free HAT-selected cells after (E) 0, 7 or 14 days proliferation (spontaneous), data are mean ± s.e.m. of 7 (7 days growth) and 2 (14 days growth) independent exp's (n =  5-50 per exp). (F) 6 days continuous treatment with 1 µM PARPi, 5 or 20 nM CPT, 1 and 4 Gy IR, Ctrl is 7 days spontaneous. Data are mean ± s.e.m. of 3 independent exps (n = 5 per exp). (G) A549 K63RUb cells were treated as described in (e) to obtain spontaneous, CPT- and IR-induced *HPRT* mutated clones. RNA and genomic DNA were isolated from individual colonies and mutations were assessed by sequence analysis of *HPRT* cDNA and/or exon1-9 presence was scored by exon-specific PCR amplification. (H) Metaphases of untreated or CPT-treated (20 nM, 24 h) WTUb and K63RUb cells were harvested. Chromosomal aberrations were scored using giemsa-staining in 50 metaphases per treatment. Data represents average ± s.e.m. *P<0.05, **P<0.01, ***P<0.001.

To examine the involvement of K63 ubiquitylation in DSB repair, we employed a strategy to selectively suppress K63-ubiquitylation, using a mutant form of Ub in which lysine 63 is replaced with arginine (K63RUb). Likewise, we overexpressed WTUb in parallel to control for potential indirect effects K63RUb overexpression might have in other ubiquitin mediated processes (Fig. 1A). Expression levels of K63RUb mRNA were ∼4 fold lower than that of endogenous ubiquitin B expression (Fig. 1B), but were sufficient to compete with WTUb and thus decrease K63-ubiquitylation (Fig. 1C) without influencing total ubiquitylation, which is mediated primarily via K48 linkage (Fig. S1A). FACS analysis indicated that overexpression of K63RUb had no effect on cell cycle distribution (Fig. 1D), in addition, proliferation of WTUb- and K63RUb expressing cells is similar (Fig. S1B). Strikingly, we observed that cells expressing K63RUb displayed a dramatic increase in spontaneous mutations at the *HPRT* locus, increasing approximately 200 fold from 1.2 mutations per 10^7^ WTUb expressing cells to 2.1 mutations per 10^5^ K63RUb expressing cells in 7 days of unperturbed growth (Fig. 1E; Fig. S1C). This increase was not caused by differences in cell proliferation (Fig. S1B). Treatment with IR or with agents that promote replication-associated DSBs by inhibiting PARP (PARPi; KU0058948) or topoisomerase I (camptothecin; CPT) resulted in a further increase in mutations in K63RUb expressing cells (Fig. 1F). These data indicate that K63RUb expressing cells experience inaccurate repair of endogenous, IR, PARPi, and CPT induced DNA damage.

K63-ubiquitylation has previously been implicated in the DNA damage tolerance pathway, which is important for response to ultraviolet (UV)-induced thymine dimers and other replication blocking lesions [26], [27]. In this pathway K63-ubiquitylation of PCNA prevents point mutations introduced by error-prone translesion polymerases [24], [27], [28], [29]. In order to determine if a similar mechanism was responsible for the increase in spontaneous and DSB-induced mutations, we analysed individual *HPRT* mutant clones. Of the spontaneous mutations 58% were single nucleotide changes, which were all G:C to T:A transversions (Fig. 1G; Table 1), consistent with endogenous 8-oxoguanine mutagenesis induced by oxidative DNA damage [30], [31]. In addition, unlike what was observed following UV damage, 32% of the spontaneous mutations were large scale deletions, and moreover the increase in mutations induced by CPT and IR was accounted for entirely by an increase in large-scale deletions (Fig. 1G; Table 1). Furthermore, PCNA is not ubiquitylated in response to DNA damage induced by CPT or IR [12], [32], [33]. To further rule out that spontaneous and CPT-induced mutations in the K63RUb cells are caused by impaired PCNA polyubiquitylation we knocked down the E3 ligases HLTF and SHPRH (Fig. S2A and B). Depletion of HLTF or SHPRH caused mutations upon treatment with UV and MMS, respectively, as previously shown [34]. Importantly depletion of HLTF or SHPRH did not lead to spontaneous or CPT-induced mutations. Thus, PCNA ubiquitylation is unlikely to be responsible for the genetic instability observed in response to S-phase DSBs in the K63RUb expressing cells.

**Table 1:** Spontaneous, IR- and CPT-induced mutation spectra in K63R ubiquitin expressing cells.

	Analysis	Point	Sequence	Position	Amino acid	Mutant	Analysis cDNA/	Deletion	Deleted	Mutant
	cDNA	mutation	change				gDNA		positions	
Spontaneous	cDNA		GAC -> TAC	579	Asp -> Tyr	SP-C	cDNA	exon 8 deletion	699–776	SP-B
	cDNA		GAC -> TAC	579	Asp -> Tyr	Sp-2	cDNA	Part of exon 7 deletion	660–700	SP-6
	cDNA		TTG -> TTT	773	Leu -> Phe	SP-4	cDNA	exon 5 deletion	552–569	SP-13
	cDNA		GAC -> TAC	579	Asp -> Tyr	SP-10	cDNA	Part of exon 8 deletion	700–720	SP-15
	cDNA	G/C -> T/A	GAC -> TAC	579	Asp -> Tyr	SP-11	cDNA	exon 5 deletion	552–569	SP-20
	cDNA		TTG -> TTT	773	Leu -> Phe	SP-19	no cDNA -> gDNA	exon 9 deletion		SP-12
	cDNA		GGA -> TGA	801	Gly -> STOP	SP-21	no cDNA -> gDNA	no exon del. detected		SP-A
	cDNA		GAC -> TAC	579	Asp -> Tyr	SP-22	no cDNA -> gDNA	no exon del. detected		SP-3
	cDNA		GAC -> TAC	579	Asp -> Tyr	SP-23				
	cDNA		GAC -> TAC	579	Asp -> Tyr	SP-24				
	cDNA		CTT -> ATT	744	Leu -> Ile	SP-17				
IR	cDNA		TTT -> TAT	709	Phe -> Tyr	IR #46	cDNA	exon 6 deletion		IR #42
	cDNA		GTG -> TTG	564	Val -> Leu	IR #48	no cDNA -> gDNA	exon 8,9 deletion		IR #26
							no cDNA -> gDNA	exon 8,9 deletion		IR #38
							no cDNA -> gDNA	exon 8,9 deletion		IR #51
							no cDNA -> gDNA	exon 8,9 deletion		IR #52
							no cDNA -> gDNA	exon 8,9 deletion		IR #53
							no cDNA -> gDNA	exon 9 deletion		IR #55
							no cDNA -> gDNA	exon 6,8,9 deletion		IR #56
							no cDNA -> gDNA	no exon del. detected		IR #36
							no cDNA -> gDNA	no exon del. detected		IR #50
CPT	cDNA		GAC -> GAA	749	Asp -> Glu	CPT #7	cDNA	exon 4,5 deletion		CPT #13
	cDNA	G/C -> T/A	GGA -> TGA	801	Gly -> STOP	CPT #15	no cDNA -> gDNA	exon 4,6,8,9 deletion		CPT #4
	cDNA		GGA -> TGA	801	Gly -> STOP	CPT #21	no cDNA -> gDNA	exon 9 deletion		CPT #5
	cDNA		GGA -> TGA	801	Gly -> STOP	CPT #26	no cDNA -> gDNA	exon 3,4,5,6,8,9 deletion		CPT #6
							no cDNA -> gDNA	exon 6 deletion		CPT #8
							no cDNA -> gDNA	exon 8,9 deletion		CPT #10
							no cDNA -> gDNA	exon 4,6,8,9 deletion		CPT #11
							no cDNA -> gDNA	exon 4 deletion		CPT #16
							no cDNA -> gDNA	exon 8,9 deletion		CPT #23
							no cDNA -> gDNA	exon 4,6,8,9 deletion		CPT #25
							no cDNA -> gDNA	exon 4,8,9 deletion		CPT #29
							no cDNA -> gDNA	exon 4,6,8,9 deletion		CPT #31
							no cDNA -> gDNA	no exon del. detected		CPT #2
							no cDNA -> gDNA	no exon del. detected		CPT #9
							no cDNA -> gDNA	no exon del. detected		CPT #14
							no cDNA -> gDNA	no exon del. detected		CPT #19
							no cDNA -> gDNA	no exon del. detected		CPT #22
							no cDNA -> gDNA	no exon del. detected		CPT #28
							no cDNA -> gDNA	no exon del. detected		CPT #30

We also assessed genomic instability by quantification of endogenous and CPT-induced chromosome aberrations (Fig. 1H). In untreated samples, K63RUb expressing cells had nearly twice as many metaphases with aberrations (18%) compared with WTUb expressing cells (10%). Following CPT treatment, 44% of metaphases from K63RUb cells displayed aberrations whereas this percentage (10%) was unchanged in WTUb cells. The number of chromosomal aberrations increased upon CPT treatment in both WTUb and K63RUb cells with 1.8- and 3.7-fold, respectively (Fig. 1H right panel). These data suggest that K63R cells experience more damage, have a DNA repair defect and/or escape cell-cycle checkpoints to migrate to mitosis with damage. Interestingly, asymmetrical radial chromosomes, which are characteristic of HR defects in BRCA1-deficient cells [35], [36], were observed exclusively in the K63RUb expressing cells (Fig. 1h). Since both spontaneous and CPT-induced collapsed replication forks are repaired primarily through HR [16], [37], these data suggest a possible requirement for K63-ubiquitylation in this pathway [16], [37].

### Disruption of K63-ubiquitylation sensitizes cells to replication-associated DNA damage

**Figure 2:**
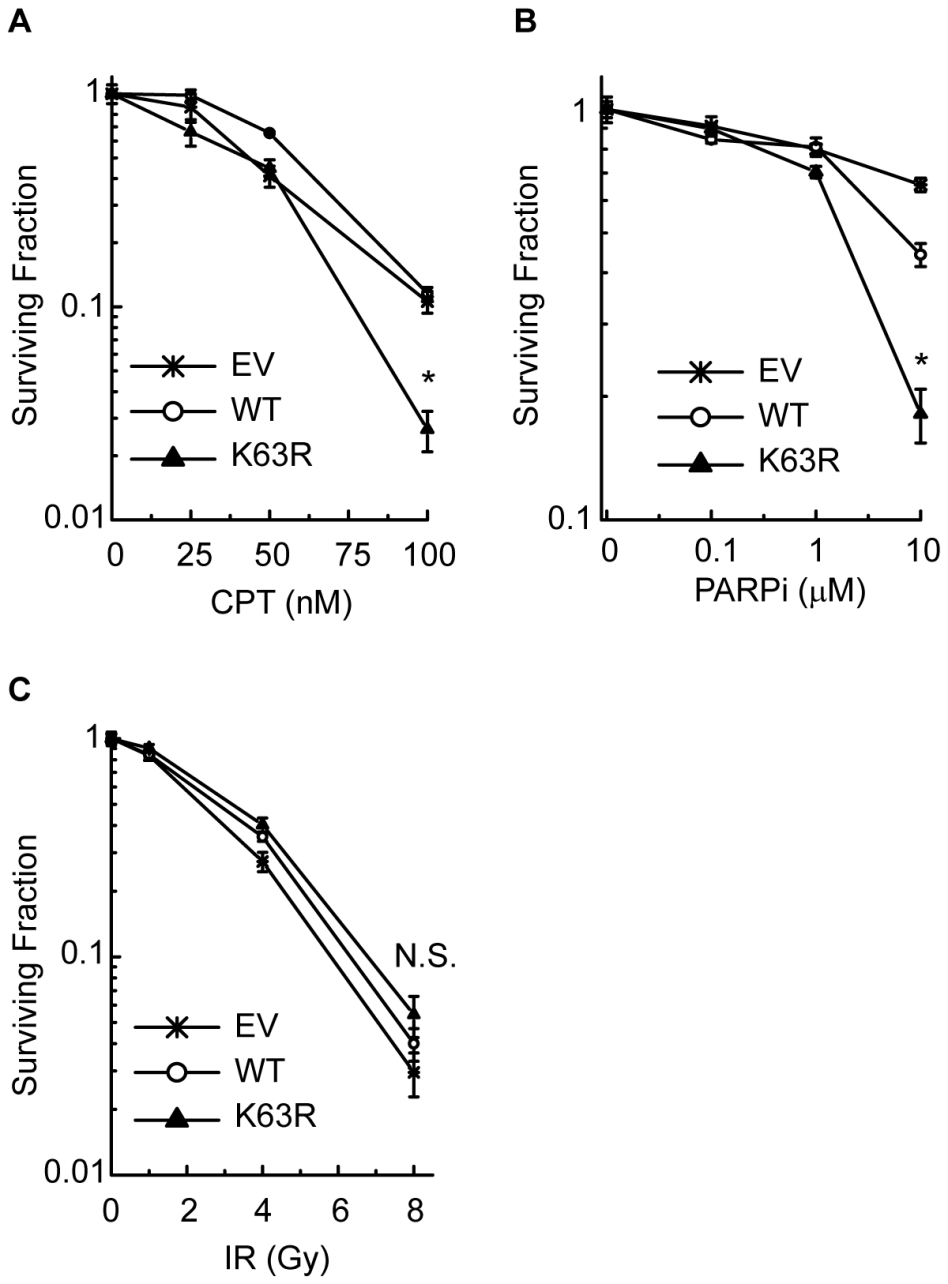
K63RUb expression sensitizes cells to replication-associated DNA damage. (A-C) Clonogenic survival of A549 cells expressing empty vector (EV), WTUb or K63RUb was determined after (A) CPT (24h) treatment started following cell attachment. Data are mean ± sd. of 2 independent exp's (n = 3 per exp). (B) Continuous PARPi treatment, data are mean ± sd. of 3 independent exp's (n = 3 per exp). (C) IR, data are mean ± sd. of 3 independent exp's (n = 3 per exp) *P<0.05, **P<0.01, ***P<0.001.

To further investigate the repair defect in cells with suppressed K63-ubiquitylation, we exposed cells to various doses of CPT, PARPi, or IR and measured clonogenic survival. HR dependent repair is known to be particularly important for sensitivity to CPT and PARPi [21], [37], [38], [39] whereas NHEJ plays a more important role in the survival of cells to IR [40]. We found that cells expressing K63RUb are markedly more sensitive to both CPT and PARPi as compared to WTUb- or empty vector (EV) overexpressing cells, but show similar sensitivity to IR (Fig. 2A, B, C). Similar results were obtained in immortalized MEFs expressing WTUb or K63RUb (Fig. S3). Knockdown of HLTF or SHPRH did not sensitize cells to CPT similarly to K63RUb expression (Fig. S2C). These data indicate that cell survival after exposure to agents that produce replication-associated DSBs is highly dependent on functional K63 ubiquitylation.

The E2 enzyme UBC13 is known to catalyse specific formation of K63-linked Ub chains in response to various types of DNA damage [5], [26], [41], [42]. To confirm our findings we depleted UBC13 in A549 cells as an independent way to interfere with K63-ubiquitylation. Partial UBC13 knockdown (Fig. S4A) was sufficient to sensitize cells to both CPT and PARPi (Fig. S4B, C), but not to IR (Fig. S4D). Together, these data indicate that disruption of K63-ubiquitylation preferentially sensitizes cells to agents that produce replication-associated DSBs. The lack of sensitivity to IR in K63RUb cells (Fig. 2C) may be due to the fact that the majority of DSBs are two-ended DSBs introduced directly from radiation damage.

### Disruption of K63-ubiquitylation results in S-phase specific defects in DNA repair

**Figure 3:**
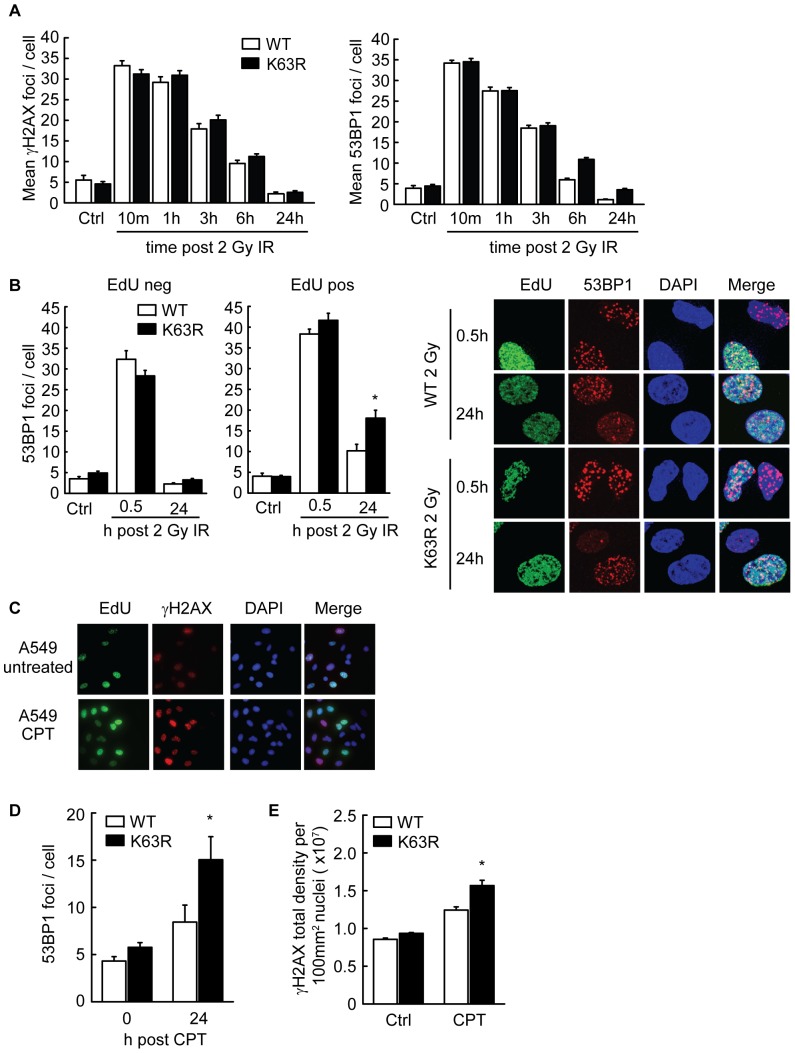
K63RUb expression induces S-phase specific repair defects. (A) WT and K63R Ub cells were fixed and immuno-stained for γH2AX and 53BP1 foci at the indicated time points following 2 Gy IR or untreated (Ctrl). Mean values ± sd. of 2 independent exp's, >150 cells were analysed per time-point (B) 30 min EdU (10 µM) incorporation before 2 Gy IR, cells were fixed 30 min or 24 hrs post IR, EdU and 53BP1 foci were visualized by fluorescent staining and quantified. Mean values ± s.e.m. of 2 independent exp's (n>100 per treatment). (C) 30 min EdU (10 µM) incorporation alone (untreated) or before 2.5 h 100 nM CPT treatment. Cells were fixed and immuno-stained for EdU and γH2AX 3 h post treatment. (D) Quantification of 53BP1 foci. Cells were fixed and immuno-stained for 53BP1 directly or 24 h after 1 h 100 nM CPT treatment, >80 cells were analysed per sample. (E) Quantification of γH2AX immuno-staining of untreated (Ctrl) or 1 h 100 nM CPT treated cells. Mean values ± s.e.m. Data is representative exp (n>130) of 3 independent exp's, *P<0.05.

To resolve the apparent discrepancy between IR-induced mutation frequency and survival observed in K63RUb cells, we assessed DNA repair kinetics at sites of damage by monitoring the formation and resolution of DNA repair foci following treatment with IR. K63-ubiquitylation has previously been shown to play an important role in the recruitment and/or retention of BRCA1 and 53BP1 to direct DSBs [5], [6], [7], [8], [9], [10], [11]. Measuring the number of γH2AX and 53BP1 foci per cell in response to IR revealed no differences in the initial recruitment of γH2AX and 53BP1 (10 min post IR) to sites of damage between WTUb and K63RUb expressing cells (Fig. 3A). However, we did observe an increase in residual 53BP1 foci measured at 6 and 24 h following IR treatment in K63RUb cells (Fig. 3A), a phenomenon that has been associated previously with defects in repair that impact on cell survival [43], [44]. The reduced 53BP1 clearance observed in K63RUb cells is also in line with slower resolution of γH2AX. It is unclear whether these residual 53BP1 foci represent unresolved direct DSBs or secondary replication-associated DSBs produced in S-phase.

To investigate whether residual damage in K63RUb cells originates from cells irradiated in S-phase, we pulse-labelled S-phase cells with EdU immediately prior to IR treatment. Analysis of residual foci in non S-phase (EdU negative) cells, or S-phase (EdU positive) cells separately, revealed that the residual 53BP1 foci were almost exclusively present in the EdU positive fraction (Fig. 3B; Fig. S5A). These results indicate that a repair defect of IR-induced lesions is limited to S-phase in K63RUb cells. To further address whether K63RUb cells are indeed compromised in their ability to repair lesions produced during replication, we treated cells with the S-phase specific DSB-inducing drug CPT for 2.5 hrs. As expected this treatment restricted damage to cells in S-phase as shown by simultaneous staining with EdU and γH2AX (Fig. 3C). Following a 1 h CPT treatment 53BP1 foci were analyzed immediately (0 h) or 24 h after treatment (Fig. 3D). Similar to the results with IR, a significant increase (p = 0.0309) in residual 53BP1 foci was observed in K63RUb cells following CPT treatment. In addition, we assessed DSB formation following treatment with CPT and PARPi using γH2AX foci formation. Interestingly, the levels of γH2AX foci in the K63RUb expressing cells were significantly increased compared to the WTUb cells (Fig. 3E; Fig. S5B), demonstrating that K63RUb cells experience more collapsed replication forks following treatment with CPT and PARPi. Altogether these data indicate that disruption of K63-ubiquitylation causes a specific defect in the repair of damage that occurs during DNA replication.

### Genetic instability following disruption of K63-ubiquitylation is not due to NHEJ

**Figure 4:**
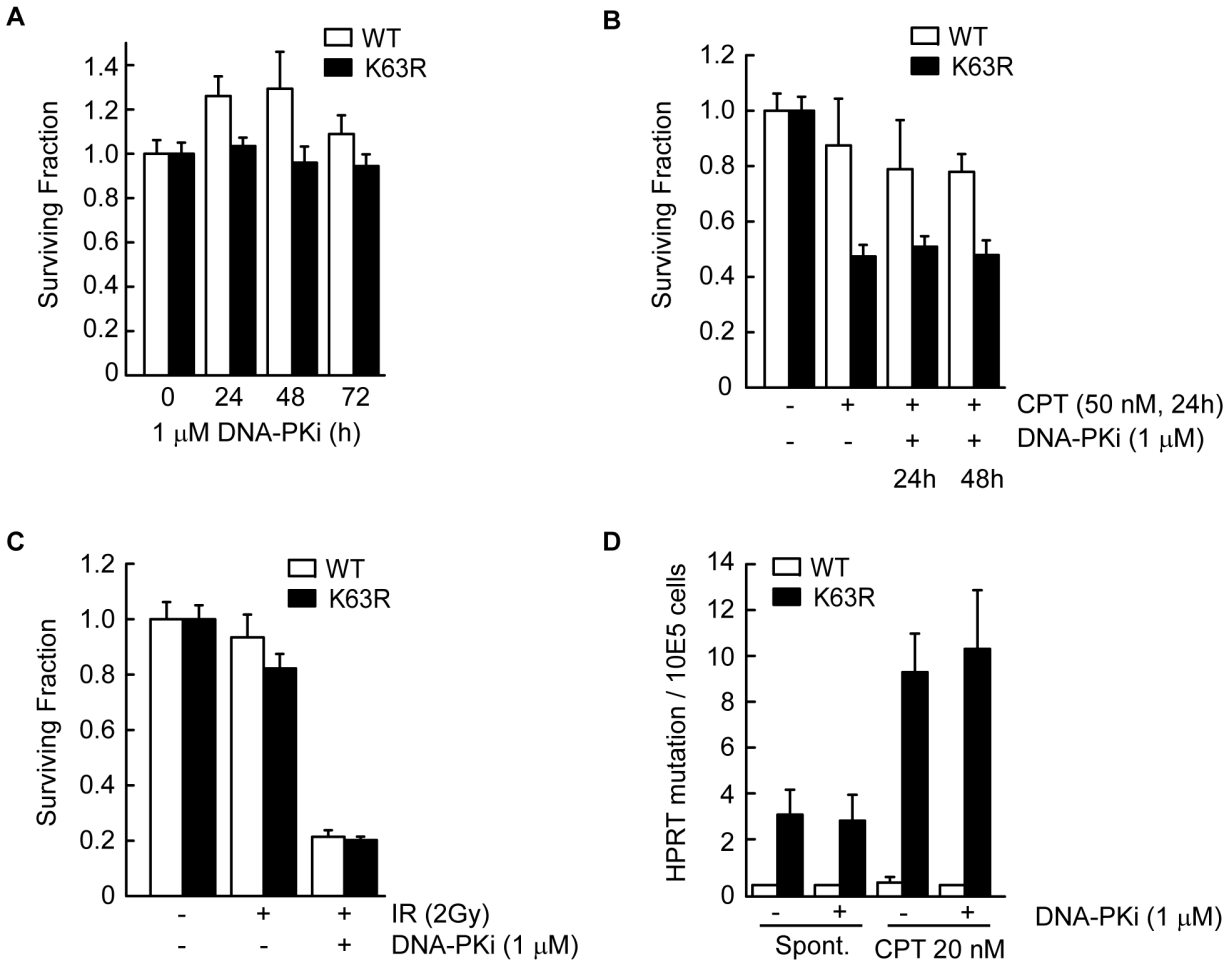
K63RUb phenotype is not due to NHEJ. (A-C) Clonogenic survival of WT and K63R Ub cells after treatment with (A) 1 µM DNA-PKi alone for indicated times, (B) 1 µM DNA-PKi for 24 or 48 hrs, started 1 h before combined 24 hrs treatment with 50 nM CPT or (C) 1 µM DNA-PKi for 24 hrs, started 1 h before 2 Gy IR (A-C) mean ± s.d. (n = 3). (D) Mutations at the *HPRT* locus were determined after continuous treatment with 1 µM DNA-PKi started 1 h before combined with additional treatments either spontaneous or 20 nM CPT for 6 days total, mean ± s.d. (n = 5).

In mammalian cells, DSBs are repaired by both error-free HR and by error-prone non-homologous end-joining (NHEJ) pathways. HR and NHEJ can compete for repair of replication-associated DSBs [40], [45], [46] and it has been suggested that NHEJ factors may have a suppressive effect on HR [47]. To determine if the increase in genetic instability and mutation rate observed following suppression of K63-ubiquitylation was due to a switch from HR to NHEJ we utilized a chemical inhibitor of DNA-dependent protein kinase catalytic subunit (DNA-PKcs) (referred to as DNA-PKi; KU0057788). Inhibition of DNA-PKcs alone had no effect on cell survival in WT or K63RUb cells (Fig. 4A), and similarly had no effect on the sensitivity to CPT (Fig. 4B). As expected, inhibition of NHEJ sensitized cells to IR, but this was equivalent in WT and K63RUb expressing cells (Fig. 4C). Furthermore, inhibition of DNA-PKcs did not rescue the increased spontaneous mutation frequency in K63RUb cells nor that induced following CPT treatment (Fig. 4D). These data indicate that K63RUb cells do not become more reliant on NHEJ for repair of replication-associated DSBs and that the genetic instability in these cells is not due to the classical NHEJ pathway.

### K63-ubiquitylation dependent repair of replication-associated DSBs is RNF8 independent

**Figure 5:**
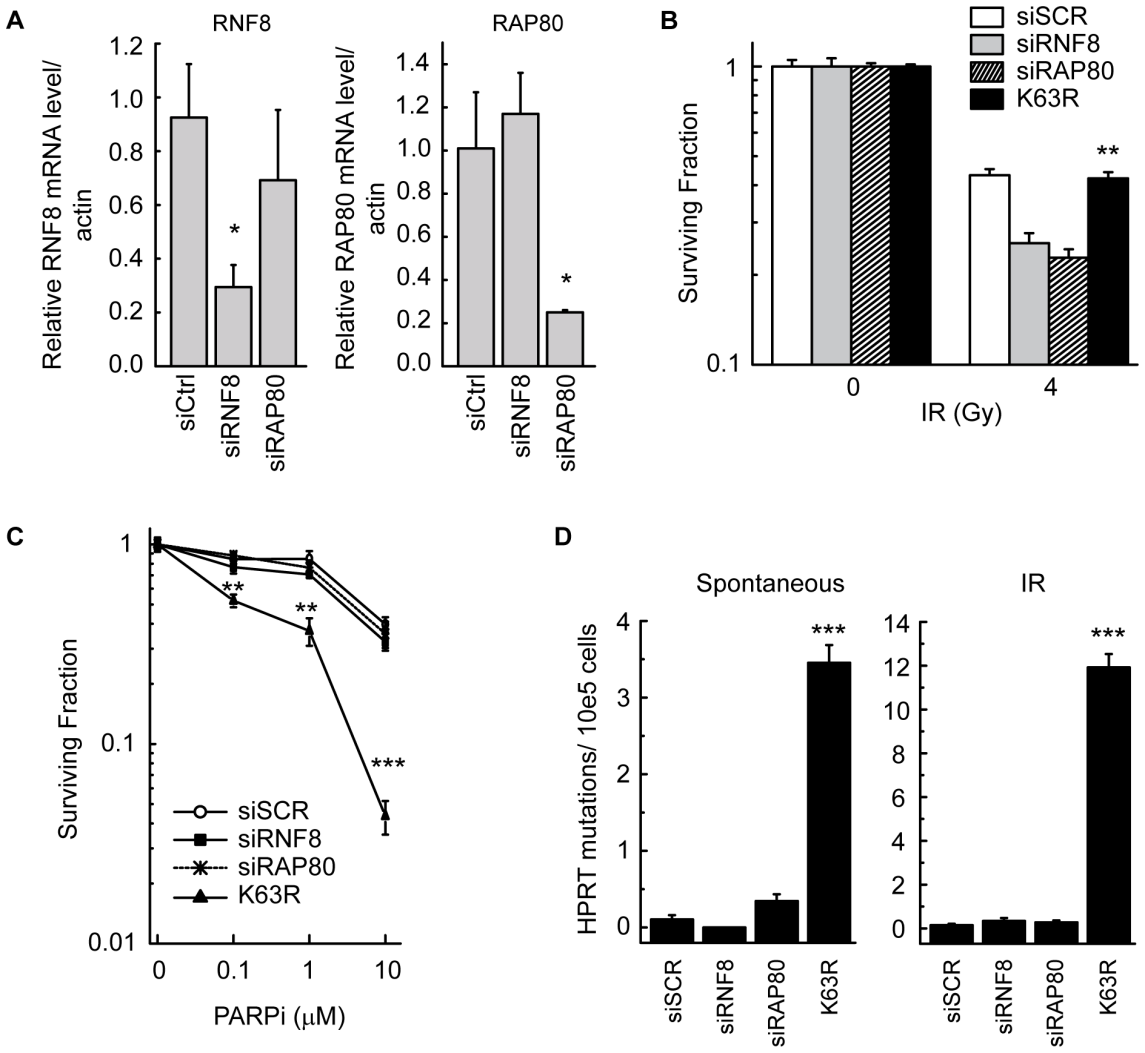
RNF8 depletion does not reproduce K63RUb phenotype. (A-C) WT and K63R Ub cells were transfected with siRNA against RNF8 or RAP80 on day 1 and 3. (A) Knockdown of RNF8 and RAP80 was assessed by mRNA expression levels determined by real-time PCR. Clonogenic survival was assessed after (B) 4 Gy IR and (C) PARPi (continuous) (B-C) Data are mean ± s.d. of 2 independent exp's (n = 3 per exp). (D) Spontaneous and IR-induced (4 Gy) mutations were determined at the *HPRT* locus, mean ± s.e.m. of 3 independent exp's (n = 10 per exp), *P<0.05, **P<0.01, ***P<0.001.

**Figure 6:**
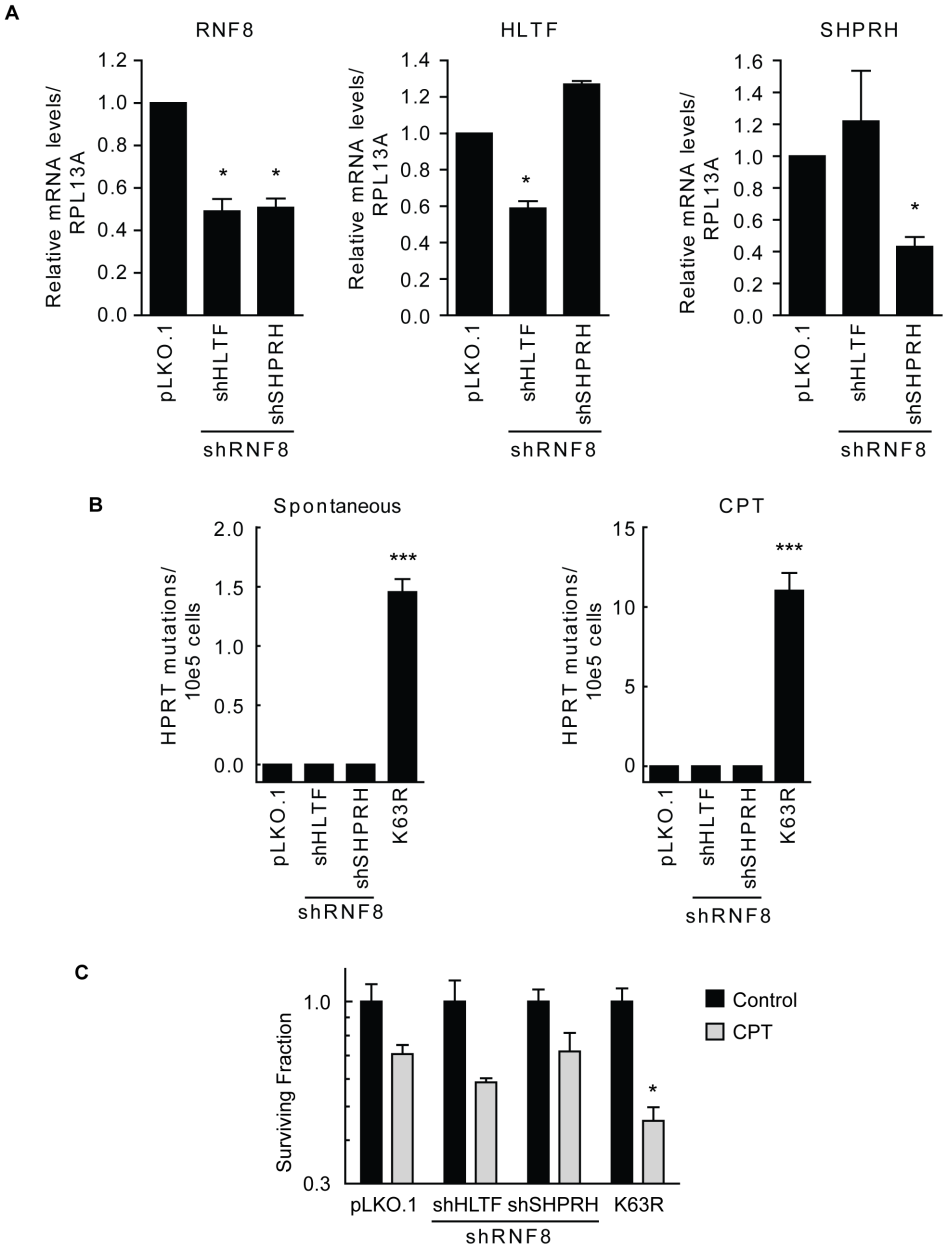
Dual inhibition of PCNA and RNF8 signalling pathways does not mimic the K63RUb phenotype. (A) Depletion of HLTF or SHPRH in combination with knock-down of RNF8 was achieved using lentiviral shRNA's and was confirmed using real-time PCR. (B) Spontaneous or CPT (20 nM) induced mutations were determined at the *HPRT* locus, mean ± s.d. (n = 5) per exp). (C) Clonogenic survival of A549 cells after double knock-down of HLTF and RNF8 or SHPRH and RNF8 was determined after treatment with 100 nM CPT (24 h). Data are mean ± s.d. (n = 3), *P<0.05, **P<0.01, ***P<0.001.

The E3 ligases RNF8 and RNF168 are known to mediate K63-ubiquitylation of H2A and γH2AX at sites of IR-induced DSBs [5], [6], [7], [8], [10], [11]. To more directly investigate their role in the response to replication-associated DSBs, we used siRNA against both RNF8 and RAP80, a downstream ubiquitin binding protein necessary for recruitment of BRCA1 to IR-induced DSBs. Knockdown of RNF8 and RAP80 following double transfection was at least 70% (Fig. 5A) and this was sufficient to sensitize WTUb expressing cells to IR to an extent similar to that reported previously, functionally validating the siRNA's used (Fig. 5B) [5], [6], [48], [49]. However, depletion of RNF8 or RAP80 did not sensitize cells to PARPi to a similar extent as that observed in cells expressing K63RUb (Fig. 5C). Consistent with these data, knock-down of RNF168 did not lead to reduced cell survival following PARP inhibition (Fig. S6). Given our observation of the importance of K63-ubiquitylation in controlling genetic stability and mutation frequency, we also assessed spontaneous and IR-induced mutations in cells following knockdown of RNF8 and RAP80. Consistent with the sensitivity data, knockdown of RNF8 or RAP80 had no measurable effect on spontaneous or IR-induced mutation frequency (Fig. 5D). These data suggest that the requirement for K63-ubiquitylation in replication-associated damage repair is independent of RNF8 or RAP80. We also investigated a scenario in which the combination of partial defects in PCNA polyubiquitylation and RNF8-dependent DSB signalling would lead to the observed phenotype in K63RUb cells. We generated cell lines with double knockdown of HLTF and RNF8 or SHPRH and RNF8 (Fig. 6A). Impairment of both signalling pathways simultaneously did not cause spontaneous or CPT-induced mutations nor did it lead to an increased sensitivity to CPT-induced replication-associated DNA damage (Fig. 6B and C). These data demonstrate that the observed K63R phenotype is not due to combined effects of partial inhibition of these two DNA repair pathways.

**Figure 7:**
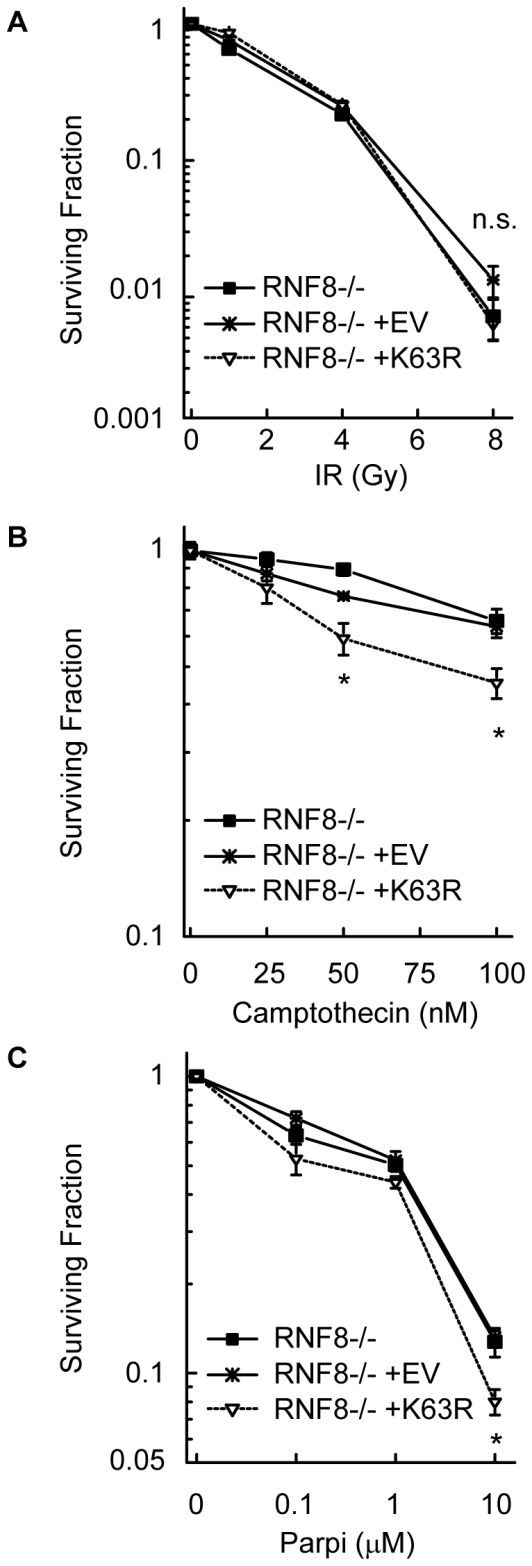
K63RUb expression sensitizes RNF8-/- MEFs to CPT and PARPi. (A-C) Clonogenic survival of RNF8-/- MEFs uninfected or infected with K63RUb or empty vector (EV) after (A) IR, (B) CPT (24 h) or (C) PARPi (continuous). Data are mean ± s.d. of 2 independent exp's (n = 3 per exp), *P<0.05.

To further confirm the RNF8-independent function of K63-ubiquitylation we performed the experiments in mouse embryonic fibroblasts (MEFs) derived from the RNF8-/- mouse [25]. Stable expression of the K63RUb transgene had no effect on cell proliferation as compared to controls (Fig. S7A). As expected, RNF8-/- cells demonstrated defective 53BP1 foci formation and increased sensitivity to IR compared to WT MEFs (Fig. S7B, C) [5], [6], [7]. Consistent with results in A549 cells, expression of K63RUb in RNF8-/- cells did not increase the sensitivity to IR (Fig. 7A) but did increase the sensitivity to both CPT and PARPi treatment (Fig. 7B, C). These data confirm a unique and critical role for K63-ubiquitylation in the response to replication-associated damage inducing agents that is independent of RNF8. Thus, although RNF8 is clearly important for K63-ubiquitylation in response to IR-induced DSBs, it is unlikely to be the major E3 responsible for K63-ubiquitylation and protection against toxicity and genetic instability in response to replication-associated DSBs.

## Discussion

K63-ubiquitylation has previously been implicated in the response to UV-induced damage, and in the response to direct DSBs produced by IR. In this study, we identify a previously unknown role for K63-ubiquitylation that contributes to the maintenance of genome stability and cell survival and which is specific for DNA replication-associated DSBs. This novel function is supported by three key pieces of evidence. First, our data demonstrate that suppression of K63-ubiquitylation through expression of the K63RUb mutant results in a dramatic increase in spontaneous mutations (>200 fold) at the *HPRT* locus (Fig. 1E; Fig. S1C). A large proportion of these mutations (∼30%) arise from large-scale deletions or other complex rearrangements. Whereas point mutations are thought to arise endogenously through mis-incorporation of single nucleotides, these endogenously produced large-scale deletions and rearrangements occur as a consequence of the misrepair of DSBs, including those at collapsed replication forks [16], [50]. The mutation rate in cells with suppressed K63-ubiquitylation was further increased in response to agents that induce DSBs. PARPi, CPT and IR increased the mutation rate in these cells by an additional 1.7-, 4.3- and 5-fold, respectively (Fig. 1F). Strikingly, this increase was due entirely to an increase in large-scale deletions, which accounted for 50-65% of the total mutations observed (Fig. 1G). Since DSBs produced by CPT and PARPi occur exclusively in S-phase as a consequence of replication fork collapse, these data imply that K63-ubiquitylation participates in their repair. The instability following suppression of K63-ubiquitylation is not specific to the *HPRT* locus, as we also observed an increase in both spontaneous and CPT-induced chromosomal aberrations (Fig. 1H) at short times after treatment. This increase is consistent with a defect in DSB repair, particularly in HR – the dominant DSB repair pathway during DNA replication, although we cannot exclude the involvement of other DNA repair mechanisms [16], [37], [51], [52], [53].

Second, we find that suppression of K63-ubiquitylation selectively increases the toxicity of replication-associated DNA damage. Cells stably expressing the K63RUb mutant are sensitive to CPT- and PARPi-induced death (Fig. 2A, B), agents that induce their cytotoxic effects through production of collapsed replication forks in S-phase [21], [22], [37]. In contrast, expression of K63RUb did not increase the cellular sensitivity to IR (Fig. 2C), which produces DSBs throughout all phases of the cell cycle and which are repaired primarily through NHEJ [54]. This result was somewhat unexpected given that RNF8 and RNF168 mediate K63-ubiquitylation of histones at IR-induced DSBs and defects in these genes cause mild sensitivity to IR [5], [6], [7], [9], [10]. However, it has been reported that the predominant form mediated by RNF8 is di-ubiquitylated γH2AX [5], [7]. It is important to note that stable expression of K63RUb suppresses, but does not entirely eliminate K63-ubiquitylation. The K63RUb mutant competes with endogenous Ub and its expression was ∼4-fold lower than endogenous ubiquitin B (Fig. 1B). This resulted in a substantial reduction, but not elimination of K63-ubiquitylation (Fig. 1C). These cells may therefore be considered ‘hypomorphic’ with respect to their ability to create these K63-Ub chains. The observation that this level of suppression selectively sensitizes cells to replication-associated DSBs implies that K63-ubiquitylation plays a more critical role in their repair as compared with IR-induced DSBs. We speculate this may be due to differences in K63-ubiquitylation chain length, as the effect of K63RUb expression will increase with chain length. In this regard, it is interesting that in contrast to IR-induced DSBs, RNF8 is unlikely to be the sole or primary mediator of K63-ubiquitylation in response to replication-associated breaks. Additional suppression of K63-ubiquitylation in RNF8 knockout cells further sensitized cells to replication-associated damage but not to IR (Fig. 7A, B, C). Importantly, RNF8 knockdown did not increase genomic instability at the *HPRT* locus (Fig. 5D). In addition, the K63R phenotype of increased mutation and sensitivity following replication-associated breaks was not due to combined effects of inhibited PCNA polyubiquitylation and defective RNF8-dependent DSB signalling (Fig. 6B and C). These disparate phenotypes support a unique role for K63-ubiquitylation in response to replication-associated DSBs and imply that a separate, as yet undetermined E3 ligase, is responsible for their formation in response to this type of damage.

Third, analysis of 53BP1 and γH2AX foci following treatment with DSB-inducing agents reveals an S-phase specific DNA repair defect in cells with impaired K63-ubiquitylation. In agreement with the lack of sensitization observed following treatment with IR, suppression of K63-ubiquitylation did not cause a general defect in establishment or resolution of 53BP1 foci at the majority of IR-induced DSBs. However, we did detect a delay in the resolution of some of these foci, as indicated by an increase in residual foci remaining at 6 and 24 hours after IR (Fig. 3A, B). Importantly, the increase in residual 53BP1 foci was observed primarily within cells that were in S-phase at the time of irradiation (Fig. 3B). Similarly, suppression of K63-ubiquitylation resulted in a significant increase in 53BP1 residual foci following CPT-treatment, which produced DSBs and 53BP1 foci exclusively in S-phase cells (Fig. 3C). Elevated levels of residual repair foci have been observed previously in cells with defects in DSB repair and their levels correlate well with cell death [43], [44], [51]. Analysis of γH2AX foci produced in response to CPT and PARPi treatment provides additional support for a defect in repair or signalling during S-phase in K63RUb expressing cells. The levels of γH2AX were significantly higher than wild-type cells at the end of CPT treatment, presumably reflecting an increase in the number of replication forks that have collapsed to produce DSBs (Fig. 3E). Together these data indicate that K63-ubiquitylation is important for the signalling and repair of DSBs produced during DNA replication. However, the large increase in mutation frequency (∼90 fold) in K63R cells following CPT treatment cannot be accounted for by increased levels of damage alone (1.3 fold increase in γH2AX foci). Thus, although there may be a small increase in damage formation, the primary defect is one in repair, and specifically repair fidelity, which leads to mutation.

The nature of the repair defect in S-phase responsible for the observed genetic instability of K63RUb expressing cells is not yet understood. Both of the primary DSB repair pathways, HR and NHEJ, can attempt repair of replication-associated lesions although HR is dominant [45], [46], [53]. Our data rules out one obvious explanation, namely that the rise in mutations is due to increased reliance of the classical, error-prone NHEJ pathway. Inhibition of the NHEJ protein DNA-PKcs resulted in strong sensitization to IR, but did not prevent mutation induction, and was not synergistically toxic in cells deficient in K63-ubiquitylation (Fig. 4A, B, C, D). However, our data do not rule out the possibility that elements of the NHEJ pathway may be involved. Recently, BRCA1 was reported to displace 53BP1 at sites of DSBs to enable HR-dependent repair [36]. The importance of this activity was demonstrated by the fact that chromosome instability and sensitivity to CPT and PARPi in BRCA1 cells was rescued by deletion of 53BP1 [36]. A model was proposed whereby 53BP1 prevented DSB end-resection necessary for HR, and instead promoted formation of aberrant chromatid fusions by Lig4 of the NHEJ pathway. Thus, 53BP1 seems to play an essential role in repair pathway choice for S-phase specific chromatid breaks. Intriguingly, suppression of K63-ubiquitylation results in a phenotype comparable to BRCA1 deficiency and BRCA1 can form a heterodimeric E3 ubiquitin ligase complex with BARD1 [55], [56], which we found was also required for resistance to PARPi (data not shown). It is thus tempting to speculate that BRCA1 may be required for K63-ubiquitylation and removal of 53BP1 at sites of collapsed replication forks. The increase in S-phase specific residual 53BP1 foci in K63RUb expressing cells (Figure 3A, B, D) supports this possibility.

These data extend the role that K63-ubiquitylation plays in maintaining genomic stability in response to S-phase specific damage, which occurs as part of at least two distinct repair pathways. In the DNA damage tolerance (DDT) pathway, K63-ubiquitylation of PCNA prevents introduction of single nucleotide mutations by error-prone translesion polymerases [24], [27]. Our findings indicate that K63-ubiquitylation plays a role in maintaining genetic stability in response to DSBs in S-phase that are associated with replication fork collapse. Further elucidation of the underlying mechanism for defective repair and identification of the ubiquitin ligases and substrates may provide potential targets to increase the efficacy of S-phase specific chemotherapeutic agents.

## Supporting Information

Figure S1 **K63RUb expression does not affect overall ubiquitylation**. (A) Western blot analysis using an antibody against ubiquitin. Ponceau S staining indicates equal loading. (B) Cell proliferation curve of A549 WTUb and K63RUb expressing cells. (C) Spontaneous mutation rate at the *HPRT* locus of WTUb and K63RUb cells on log-scale to visualize the difference.
(TIF)

Figure S2 **Depletion of E3 ligases HLTF and SHPRH does not reproduce the K63RUb phenotype.** (A) Knock-down of HLTF or SHPRH using lentiviral shRNA was confirmed by real-time PCR. (B) Spontaneous, CPT- (20 nM), UV- (20 J/m2) or MMS- (2 µg/ml) induced mutations were determined at the *HPRT* locus, mean ± s.d. (n = 5). (C) Clonogenic survival of A549 cells expressing an shRNA against HLTF, SHPRH or empty vector (pLKO.1) was determined after treatment with 100 nM CPT (24 h). Data are mean ± s.d. (n = 3).
(TIF)

Figure S3 **K63RUb expression sensitizes WT MEFs to DNA damage in S-phase**. (A-C) Clonogenic survival of WT MEFs expressing WTUb or K63RUb was determined after (A) CPT (24 h) treatment started following cell attachment, (B) continuous PARPi treatment, (C) IR. (A-C) Data are mean ± s.d. of 2 independent exp's (n = 3 per exp).
(TIF)

Figure S4 **Loss of UBC13 sensitizes to replication-associated DSBs.** (A) Knock-down of UBC13 using lentiviral shRNA was confirmed by real-time PCR. (B-D) Clonogenic survival of A549 cells expressing empty vector (pLKO.1) or shRNA against UBC13 was determined after (B) CPT (24 h) treatment started following cell attachment. Data are mean ± sd. of 2 independent exp's (n = 3 per exp). (C) continuous PARPi treatment, data are mean ± sd. of 2 independent exp's (n = 3 per exp). (D) IR, data are mean ± sd. of 2 independent exp's (n = 3 per exp).
(TIF)

Figure S5 (A) Enlarged immunostaining images shown in Fig. 3b of the manuscript (B) Quantification of γH2AX immuno-staining in WTUb and K63RUb cells treated for 6 and 24 hrs with 1 µM PARPi. Mean values ± s.e.m. of representative exp (n>100 per treatment).
(TIF)

Figure S6 **K63R induced PARPi sensitivity is not mediated by RNF168.** (A) Knock-down of RNF168 using siRNA was confirmed by WB. Actin was used as loading control. (B) Clonogenic survival of A549 cells transfected with siRNF8, siControl or untransfected was determined after continuous PARPi treatment, data are mean ± sd. of 2 independent exp's (n = 3 per exp).
(TIF)

Figure S7 **Validation of WT MEFs and RNF8-/- MEFs**. (A) Proliferation curve of uninfected RNF8-/- MEFs and lentiviral infected RNF8-/- MEFs expressing empty vector (EV) or K63RUb. (B) Clonogenic survival of WT MEFs and RNF8-/- MEFs following IR. Data are mean ± sd. of 2 independent exp's (n = 3 per exp). (C) WT MEFs and RNF8-/- MEFs uninfected or expressing EV or K63RUb cells were fixed and immuno-stained for γH2AX and 53BP1 foci following 2 Gy 30 min. Infection of RNF8-/- cells with the different constructs did not affect the defect in 53BP1 foci formation.
(TIF)
