# Supplementary figures and images for: RNF8-Independent Lys63 Poly-Ubiquitylation Prevents Genomic Instability in Response to Replication-Associated DNA Damage
Source: PLoS One. 2014 Feb 28;9(2):e89997. doi: 10.1371/journal.pone.0089997 (PMC3938561; doi:10.1371/journal.pone.0089997)

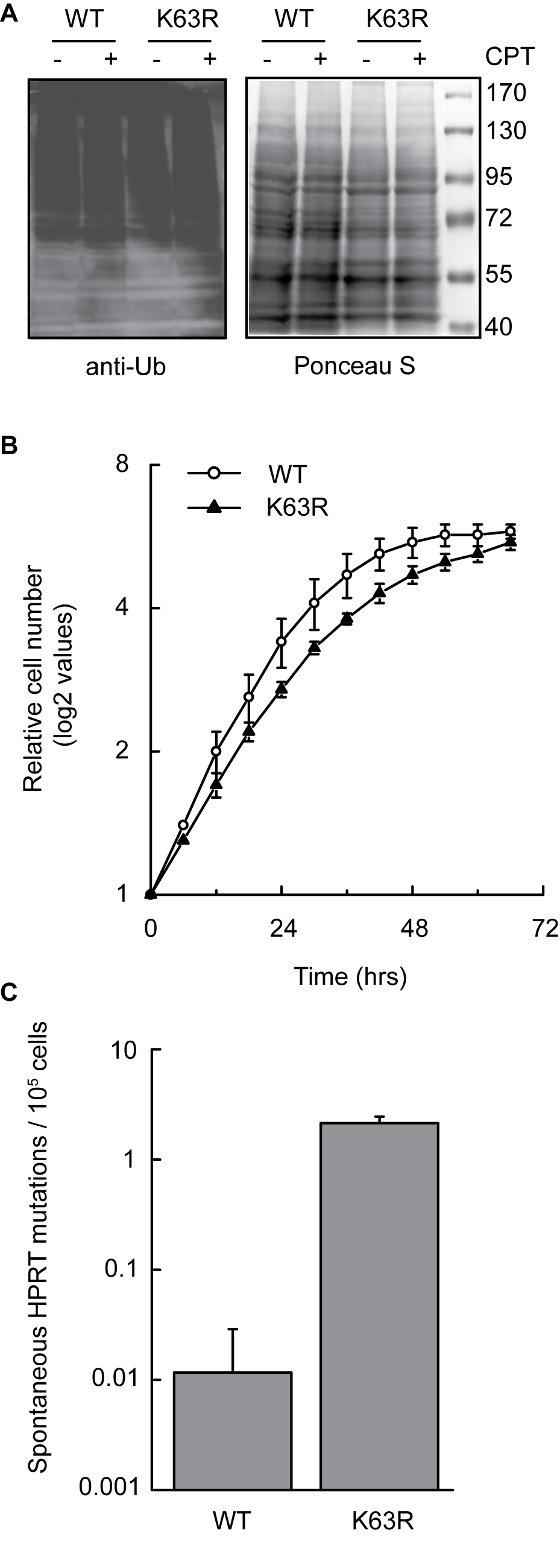

Supplement: Figure S1 — K63RUb expression does not affect overall ubiquitylation. (A) Western blot analysis using an antibody against ubiquitin. Ponceau S staining indicates equal loading. (B) Cell proliferation curve of A549 WTUb and K63RUb expressing cells. (C) Spontaneous mutation rate at the HPRT locus of WTUb and K63RUb cells on log-scale to visualize the difference. (TIF) [file pone.0089997.s001.tif]

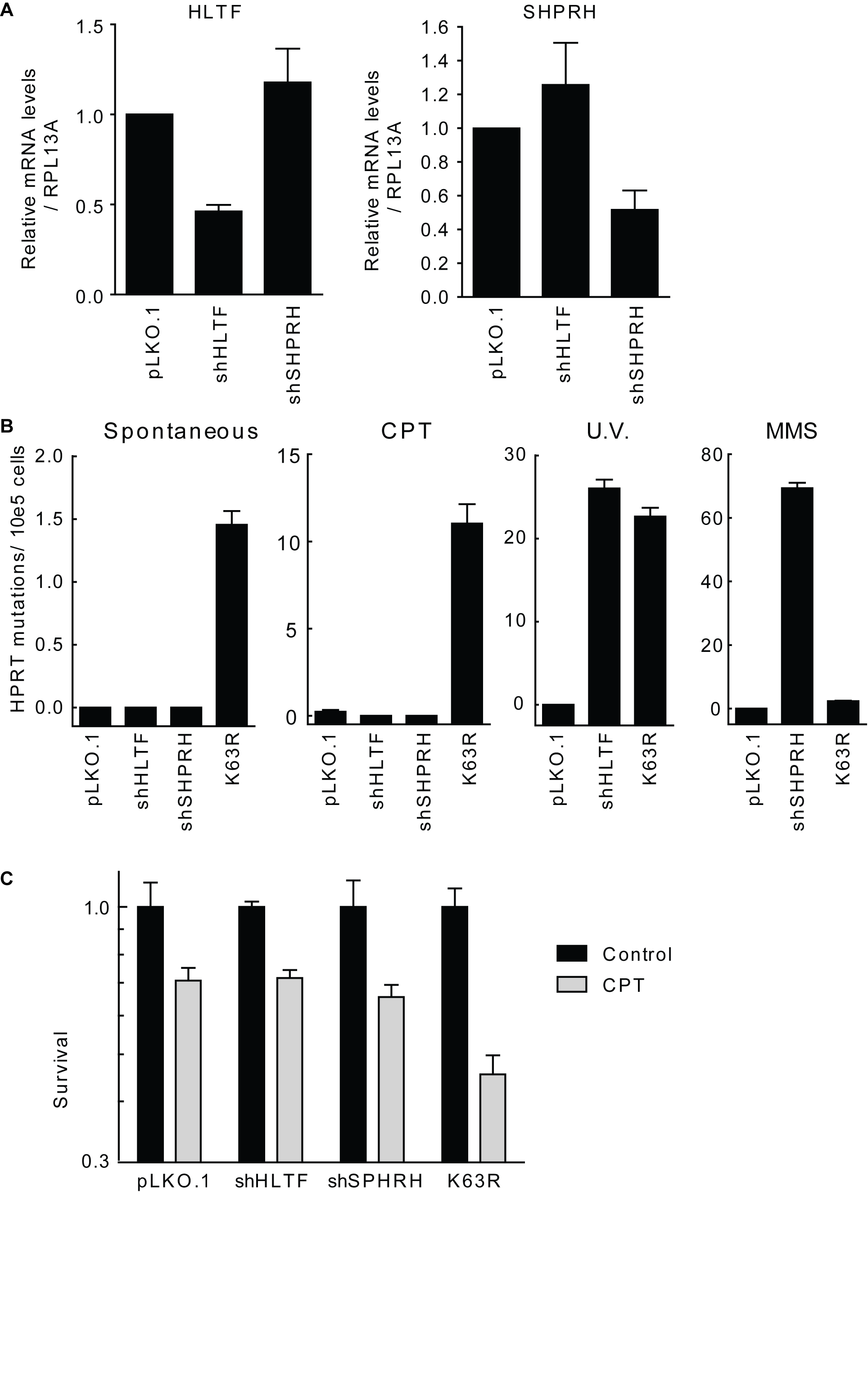

Supplement: Figure S2 — Depletion of E3 ligases HLTF and SHPRH does not reproduce the K63RUb phenotype. (A) Knock-down of HLTF or SHPRH using lentiviral shRNA was confirmed by real-time PCR. (B) Spontaneous, CPT- (20 nM), UV- (20 J/m2) or MMS- (2 µg/ml) induced mutations were determined at the HPRT locus, mean ± s.d. (n = 5). (C) Clonogenic survival of A549 cells expressing an shRNA against HLTF, SHPRH or empty vector (pLKO.1) was determined after treatment with 100 nM CPT (24 h). Data are mean ± s.d. (n = 3). (TIF) [file pone.0089997.s002.tif]

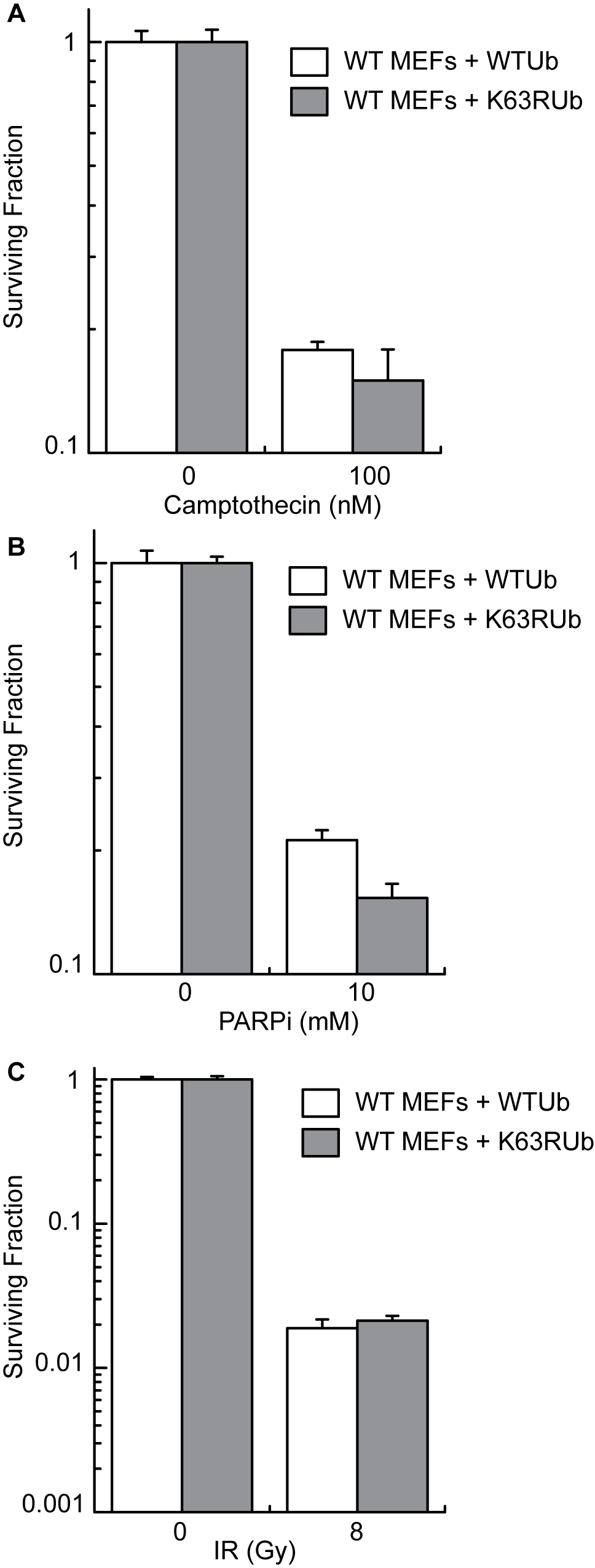

Supplement: Figure S3 — K63RUb expression sensitizes WT MEFs to DNA damage in S-phase. (A-C) Clonogenic survival of WT MEFs expressing WTUb or K63RUb was determined after (A) CPT (24 h) treatment started following cell attachment, (B) continuous PARPi treatment, (C) IR. (A-C) Data are mean ± s.d. of 2 independent exp's (n = 3 per exp). (TIF) [file pone.0089997.s003.tif]

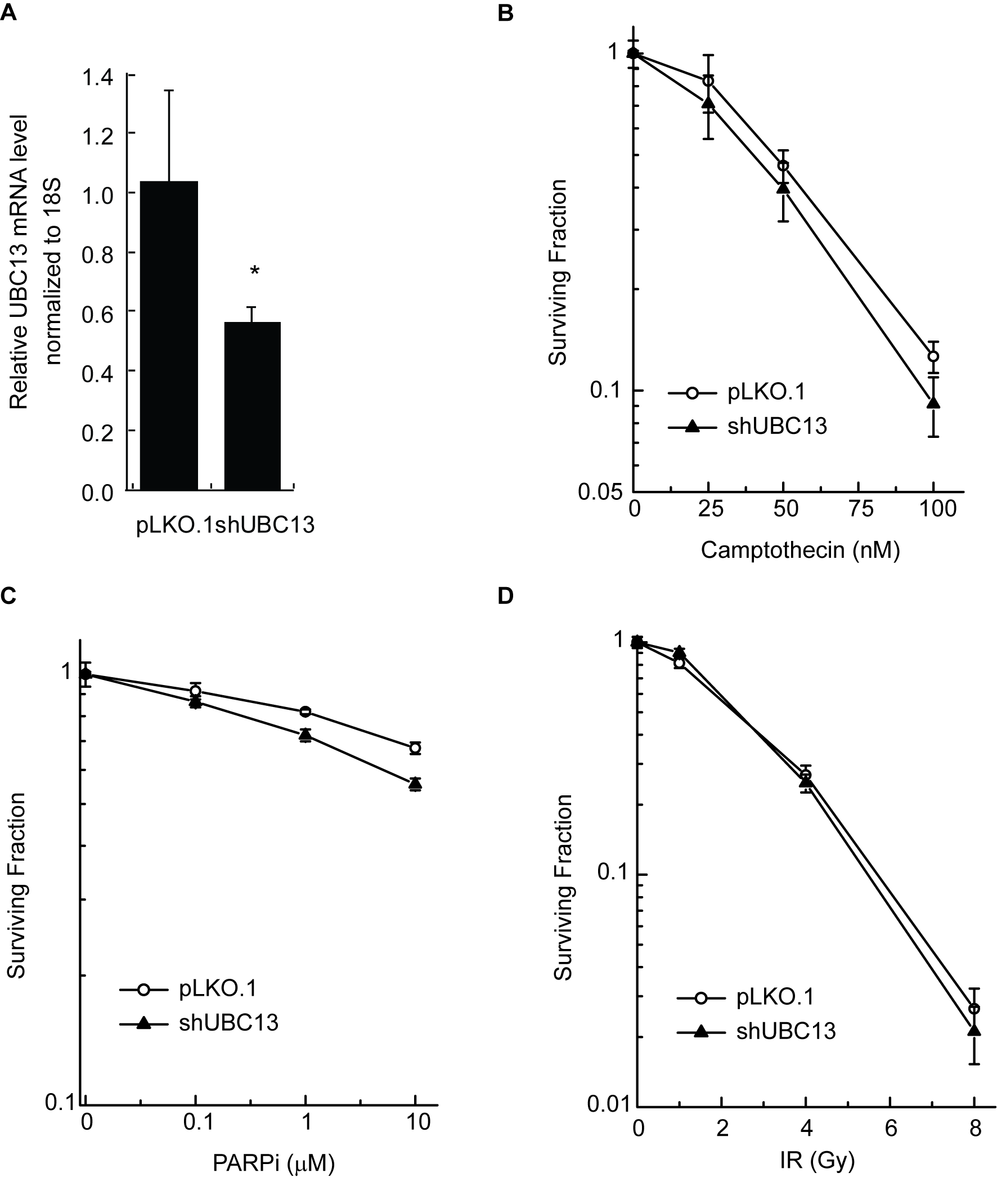

Supplement: Figure S4 — Loss of UBC13 sensitizes to replication-associated DSBs. (A) Knock-down of UBC13 using lentiviral shRNA was confirmed by real-time PCR. (B-D) Clonogenic survival of A549 cells expressing empty vector (pLKO.1) or shRNA against UBC13 was determined after (B) CPT (24 h) treatment started following cell attachment. Data are mean ± sd. of 2 independent exp's (n = 3 per exp). (C) continuous PARPi treatment, data are mean ± sd. of 2 independent exp's (n = 3 per exp). (D) IR, data are mean ± sd. of 2 independent exp's (n = 3 per exp). (TIF) [file pone.0089997.s004.tif]

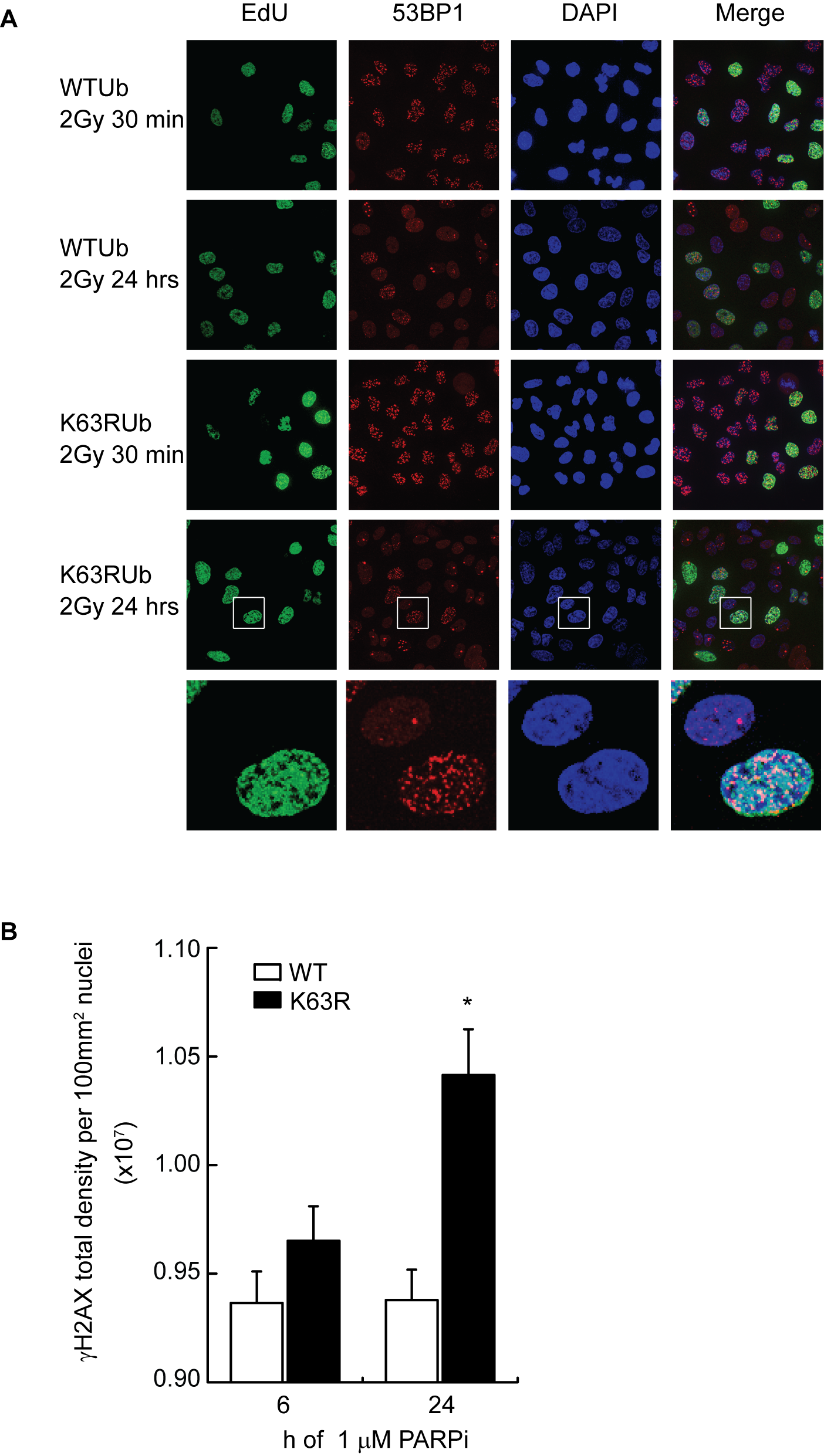

Supplement: Figure S5 — (A) Enlarged immunostaining images shown in Fig. 3b of the manuscript (B) Quantification of γH2AX immuno-staining in WTUb and K63RUb cells treated for 6 and 24 hrs with 1 µM PARPi. Mean values ± s.e.m. of representative exp (n>100 per treatment). (TIF) [file pone.0089997.s005.tif]

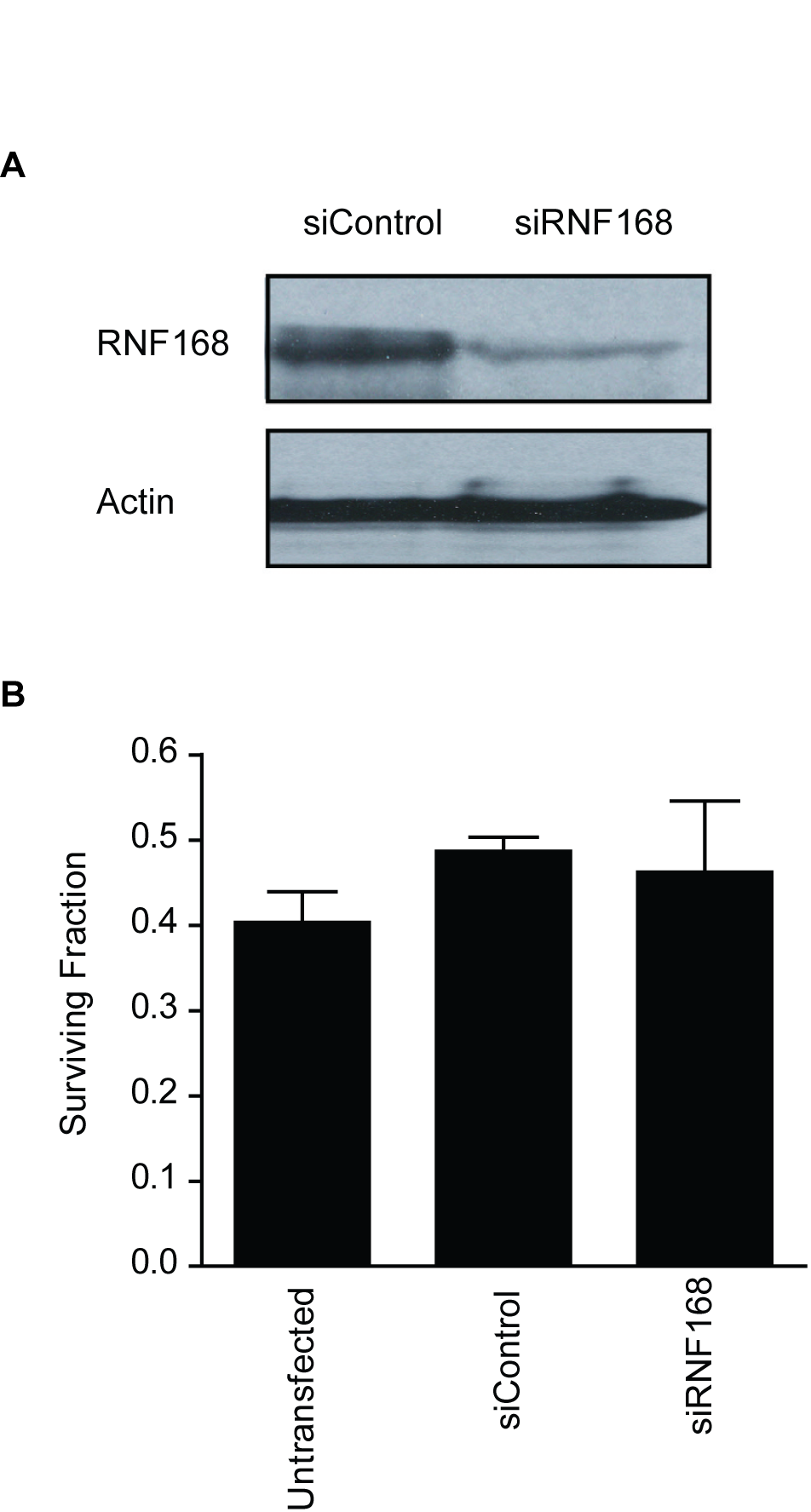

Supplement: Figure S6 — K63R induced PARPi sensitivity is not mediated by RNF168. (A) Knock-down of RNF168 using siRNA was confirmed by WB. Actin was used as loading control. (B) Clonogenic survival of A549 cells transfected with siRNF8, siControl or untransfected was determined after continuous PARPi treatment, data are mean ± sd. of 2 independent exp's (n = 3 per exp). (TIF) [file pone.0089997.s006.tif]

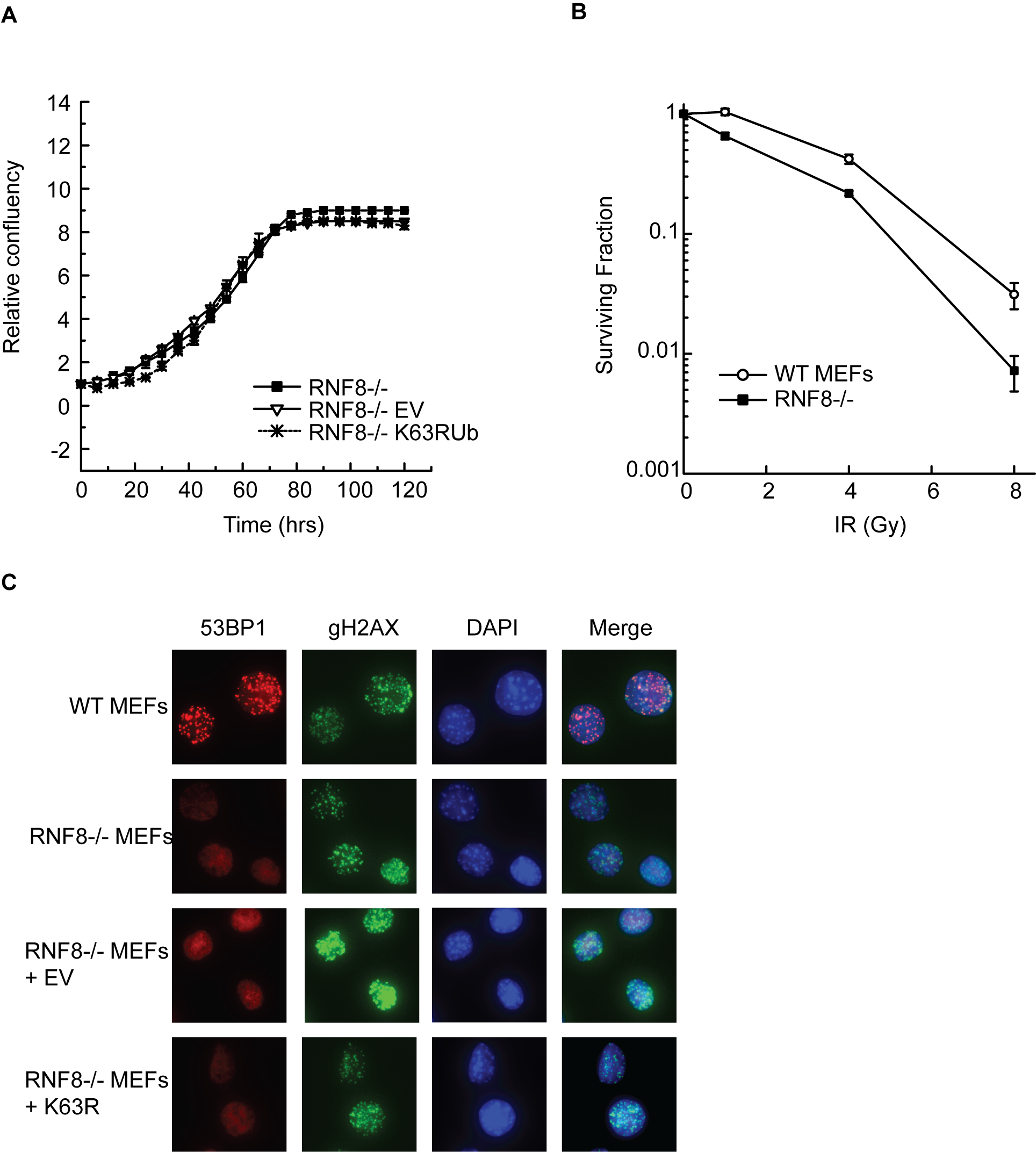

Supplement: Figure S7 — Validation of WT MEFs and RNF8-/- MEFs. (A) Proliferation curve of uninfected RNF8-/- MEFs and lentiviral infected RNF8-/- MEFs expressing empty vector (EV) or K63RUb. (B) Clonogenic survival of WT MEFs and RNF8-/- MEFs following IR. Data are mean ± sd. of 2 independent exp's (n = 3 per exp). (C) WT MEFs and RNF8-/- MEFs uninfected or expressing EV or K63RUb cells were fixed and immuno-stained for γH2AX and 53BP1 foci following 2 Gy 30 min. Infection of RNF8-/- cells with the different constructs did not affect the defect in 53BP1 foci formation. (TIF) [file pone.0089997.s007.tif]
